# Chimeric Antigen Receptor T Cells With Modified Interleukin-13 Preferentially Recognize IL13Rα2 and Suppress Malignant Glioma: A Preclinical Study

**DOI:** 10.3389/fimmu.2021.715000

**Published:** 2021-11-08

**Authors:** Kiwan Kim, Ho-Shin Gwak, Nayoung Han, Eun Kyung Hong, Beom K. Choi, Sangeun Lee, Soyoung Choi, Ju-Hwang Park, Ji-Hye Seok, Yeongha Jeon, Hyuntae Cho, Song-Jae Lee, Yura Lee, Ki Taek Nam, Seong-Won Song

**Affiliations:** ^1^ Department of Drug Development I, CellabMED Inc., Seoul, South Korea; ^2^ Department of Cancer Biomedical Science, National Cancer Center Graduate School of Cancer Science and Policy, Goyang, South Korea; ^3^ Department of Pathology, Program for Immunotherapy Research, National Cancer Center, Goyang, South Korea; ^4^ Biomedicine Production Branch, Program for Immunotherapy Research, National Cancer Center, Goyang, South Korea; ^5^ Department of Process Development, CellabMED Inc., Seoul, South Korea; ^6^ Department of Drug Development II, CellabMED Inc., Seoul, South Korea; ^7^ Department of Clinical Development, CellabMED Inc., Seoul, South Korea; ^8^ Research Institute, CellabMED Inc., Seoul, South Korea; ^9^ Severance Biomedical Science Institute, Brain Korea 21 PLUS Project for Medical Science, Yonsei University College of Medicine, Seoul, South Korea; ^10^ CellabMED Inc., Seoul, South Korea

**Keywords:** chimeric antigen receptor T cell, immunohistochemistry, interleukin-13, malignant glioma, immunotherapy

## Abstract

**Background:**

Interleukin-13 receptor α 2 (IL13Rα2) is a promising tumor-directed antigen of malignant glioma (MG). Here, we examine the efficacy and safety of T cells containing a YYB-103 chimeric antigen receptor (CAR) that can preferentially bind to IL13Rα2 on MG cells.

**Methods:**

IL13 was modified on the extracellular domain by substitution of amino acids with E13K, R66D, S69D, and R109K and stably transfected into human T cells using a retroviral vector. The *in vitro* efficacy of YYB-103 CAR T cells was tested in cell lines with differing IL13Rα1 and IL13Rα2 expression. The *in vivo* efficacy of intracerebroventricular (i.c.v.) and intravenous (i.v.) routes of YYB-103 CAR T-cell administration were tested in orthotopic MG mouse models. Immunohistochemical staining of MG was performed using WHO grade 3/4 surgical specimens from 53 patients. IL13Rα2 expression was quantified by H-score calculated from staining intensity and percentage of positive cells.

**Results:**

Binding affinity assay of YYB-103 verified apparently nil binding to IL13Rα1, which was more selective than previously reported IL13 modification (E13Y). YYB-103 CAR T cells showed selective toxicity toward co-cultured U87MG (IL13Rα1^+^/IL13Rα2^+^) cells but not A431 (IL13Rα1^+^/IL13Rα2^−^) cells. Consistently, YYB-103 CAR T cells suppressed tumor growth in nude mice receiving orthotopic injection of U87 MG cells. Both i.c.v. and i.v. injections of YYB-103 CAR T cells reduced tumor volume and prolonged overall survival of tumor-bearing mice. The median H-score for IL13Rα2 in patient-derived MG tissue was 5 (mean, 57.5; SD, 87.2; range, 0 to 300).

**Conclusion:**

This preclinical study demonstrates the efficacy of IL13Rα2-targeted YYB-103 CAR T cells against MG cells. The use of modified IL13 to construct a CAR facilitated the selective targeting of IL13Rα2-expressing MG cells while sparing IL13Rα1-expressing cells. Notably, YYB-103 CAR T cells exhibited effective blood–brain barrier crossing, suggesting compatibility with i.v. administration rather than intracranial injection. Additionally, the high H-score for IL13Rα2 in glioblastoma, especially in conjunction with the poor prognostic markers of wild-type isocitrate dehydrogenase-1 (IDH-1) and unmethylated *O* ^6^-methyl guanine methyl-transferase (MGMT), could be used to determine the eligibility of patients with recurrent glioblastoma for a future clinical trial of YYB-103 CAR T cells.

## Introduction

Malignant glioma (MG) is a common and devastating primary brain tumor that leads to death in most cases (1). Due to the infiltrating nature of MG, the current treatment regimen of maximal cytoreductive surgery followed by chemo-/radiotherapy rarely achieves long-term survival despite improving outcomes to some extent (2). Thus, the development of specific immunotherapies against targeted tumor cells is a promising approach to treating MG (3). However, the recent failure of clinical trials of check-point inhibitors in glioblastoma (GBM) raises questions about the efficacy of immunotherapy for this so-called “cold tumor,” which has low mutational burden and a paucity of tumor-infiltrating lymphocytes (4). Therefore, developing tumor-directed antigens of MG is a key step for immune-based cellular therapies. Interleukin-13 receptor α2 (IL13Rα2) is a promising target due to its abundant and specific expression in MG relative to low-grade glioma or normal brain tissue (5–8). IL13Rα2 is expressed in ~40%–60% of GBM patients and rarely in normal cells, except for those in the testicle (8, 9). IL13 binds with IL13Rα1 with low affinity and forms a heterodimer complex with IL4R that activates the Jak/STAT6 signaling pathway, resulting in downstream signaling activation (10). However, when IL13Rα2 is expressed, IL13 binds to IL13Rα2 with higher affinity, thereby inhibiting IL13Rα1/IL4R signaling. Such a mechanism is known to induce tumor metastasis and inhibit apoptosis, thereby inducing tumor malignancy. Moreover, IL13Rα2 expression in patients is correlated with a low survival rate (11). Several IL13Rα2-targeting therapies, including chimeric antigen receptor (CAR) T cells targeting IL13Rα2, IL13Rα2-targeted immunotoxins, IL13-expressing virus, anti-IL13Rα2 antibody therapy, and IL13Rα2-targeted tumor vaccine, have been tested in clinical trials and found to be safe (11–14).

The cutting-edge technology of CAR T cells employs genetic engineering to achieve ligand specificity with a high-affinity single-chain fragment variable for a target of interest and lethality from full activation and co-stimulation of fused domains (15). By bypassing antigen presentation and processing by dendritic cells or macrophages, CAR T cells are expected to overcome the challenge of low antigenicity in GBM (3, 15). Preclinical studies using IL13Rα2-specific CAR T cells show positive outcomes following direct administration to the brain, such as *via* intratumoral, intracavity, or intracerebroventricular (i.c.v.) infusion (16–19). Furthermore, a recent clinical trial of IL13Rα2-targeting CAR T cells delivered *via* i.c.v. infusion achieved a complete response of cerebrospinal fluid contact lesions (13).

Here, we aimed to verify the efficacy of newly developed YYB-103, an IL13Rα2-targeting CAR T cell using modified IL13 as an antigen-binding domain to lower the binding affinity for IL13Rα1 expressed in normal cells, using both *in vitro* and *in vivo* models, and we explored the possibility of its intravenous (i.v.) administration. We also evaluated IL13Rα2 expression in tissues samples from Korean MG patients and its association with various clinical variables and molecular markers as preliminary evidence for a future clinical trial.

## Materials and Methods

YYB-103 CAR T-cell production using human blood was approved by the Institutional Review Board (IRB) of the Korea National Institute for Bioethics Policy (P1-201510-31-005). The retrospective use of human MG and normal tissue was approved by the IRB of the National Cancer Center (NCC2020-0134), which waived the need for informed consent. All study protocols abided by the precepts established by the Declaration of Helsinki.

### DNA Constructs

YYB-103, an IL13Rα2-specific CAR with mutant IL13.E13K.R66D.S69D.R109K derived from human IL13 (GenBank no. AAH96141.2), human CD8 hinge, human CD8 transmembrane domain (GenBank no. AAH25715.1), human 41BB cytoplasmic domain (NCBI Reference Sequence. NP_001552.2), and human CD3ζ (GenBank no. AAH25703.1) was synthesized and subcloned into an MFG-based retroviral vector using *Xho*I and *Not*I sites (17).

### Surface Plasmon Resonance

A wild-type (WT) IL13 protein and an antigen binding domain of YYB-103 (YYB-103 IL13) were purified for affinity measurements. Binding affinities of WT-IL13 and YYB-103 IL13 were measured using the Biacore T200 molecular interaction system (GE Healthcare, Chicago, IL, USA). CM5 sensor chips were activated for immobilization with 1-ethyl-3-(3-dimethylaminopropyl)-carbodiimide hydrochloride and *N*-hydroxysuccinimide. Human IL13Rα1 and IL13Rα2 in 10 mM of sodium acetate (pH 4.5) were immobilized at a density of 2,800 resonance units for IL13Rα1 and 3,100 resonance units for IL13Rα2 in two flow cells on separate sensor chips. Sensor chips were then deactivated with 1 M of ethanolamine hydrochloride (pH 8.5). Purified wt-IL13 and YYB-103 IL13 were injected over the chip with a constant flow rate (30 ml/min) in twofold serial dilutions. Association and dissociation rates were monitored for 180 and 600 s, respectively. Sensor chips were regenerated with 10 mM of glycine (pH 2.0). Data were fit using a 1:1 fitting model with Biacore T200 evaluation software (Control software version 2.0.1 and BIA evaluation software version 3.0).

### Retrovirus Production

Plasmid DNA encoding YYB-103 was used to transfect a mixture of Phoenix Ampho and Phoenix Eco cells. Transduction was performed on PG13 cells using viral supernatant obtained from the cell mixture. One clone showing the highest transduction efficiency was selected through single cell sorting and produced as a cGMP-quality master cell bank. The viral supernatant was obtained from the master cell bank and produced as a cGMP quality master virus bank, which was used to introduce YYB-103 into T cells.

### YYB-103 Chimeric Antigen Receptor T-Cell Production

Human peripheral blood mononuclear cells were isolated by density gradient centrifugation using a Ficoll-Paque (GE Healthcare). Cells were stimulated with 500 IU/ml of IL2 (Novartis, Basel, Switzerland), 100 ng/ml of anti-human CD3 antibody (eBioscience, San Diego, CA, USA), and 100 ng/ml of anti-human CD28 antibody (eBioscience) for 48 h. The retrovirus encoding YYB-103 was incubated for 2 h on six-well plates coated with RetroNectin (Clontech, Mountain View, CA, USA), and stimulated cells were added to the retrovirus-coated well and incubated for 24 h for transduction. Cells were maintained at 37°C and 6% CO_2_ in cultured media for 9 days. Activated untransduced (UnTd) T cells from the same donors were used as controls in all experiments.

### Western Blotting

Tunicamycin (Sigma, St. Louis, MO, USA) at 5 μg/ml was added to T-cell cultures 24 h before harvesting cells. After cell lysis using radioimmunoprecipitation assay (RIPA) buffer containing a protease inhibitor (Thermo Fisher, Waltham, MA, USA) and EDTA (Thermo Fisher), a bicinchoninic acid (BCA) protein assay kit (Thermo Fisher) was used to measure the amount of protein. Cell lysates (20 μg/ml) were electrophoresed on NuPAGE 4%–12% Bis-Tris Gel (Invitrogen, Carlsbad, CA, USA) and transferred to a polyvinylidene difluoride membrane. Membranes were blocked for 1 h in Tris-buffered saline with 0.5% Tween-20 (TBST) and 5% skim milk. Anti-human CD3ζ antibodies (BD Biosciences, Franklin Lakes, NJ, USA) were diluted at 0.25 μl/ml in TBST and incubated with the membrane overnight at 4°C. Blots were washed with TBST and incubated with affinity purified antibody peroxidase-labeled goat anti-mouse IgG human serum adsorbed liquid conjugate (SeraCare, Milford, MA, USA) for 1 h at room temperature. Proteins were detected by incubation with enhanced chemiluminescence (ECL) Western blotting substrate (Thermo Fisher) and exposure to X-ray film.

### Flow Cytometric Analysis

Cells were harvested and resuspended in phosphate-buffered saline (PBS) containing 1% fetal bovine serum (FBS). Cell suspensions (5 × 10^5^ cells/sample) were stained with primary labeled antibodies according to the manufacturer’s instructions and incubated for 1 h at 4°C. After cells were washed with PBS, flow cytometry was performed using a BD FACSCanto™ II cell analyzer (BD Biosciences). U87, A431, Hek293FT, and Hek293FT_IL13Rα2 cells were analyzed with anti-human IL13Rα2–fluorescein isothiocyanate (FITC) antibody (R&D Systems, Minneapolis, MN, USA) and anti-human IL13Rα1-FITC antibody (R&D Systems). YYB-103 and UnTd T cells were analyzed with anti-human IL13-PE (BD Biosciences) for IL13 expression. Isotype antibodies were used as negative controls in all experiments.

### Cytotoxicity Assay

Target cells (10,000 cells/well) and effector cells were mixed according to the indicated effector-to-target (E:T) cell ratio and co-cultured in a V-bottom 96-well plate at 37°C for 18 h in a CO_2_ incubator. The cytotoxicity of YYB-103 was determined by a CytoTox96 non-radioactive cytotoxicity assay measuring the release of lactate dehydrogenase (Promega, G1780, Madison, WI, USA) according to the manufacturer’s instructions.

### Cytokine Production Assays

Effector cells (1 × 10^5^ cells/well) were co-cultured with an equal number of target cells in a 96-well plate at 37°C for 18 h in CO_2_ incubator. IFN-γ assay was performed using cell supernatant with a human IFN-γ ELISA kit (R&D System) according to the manufacturer’s instructions. The amounts of granulocyte-macrophage colony-stimulating factor (GM-CSF), IL2, IL4, and IFN-γ were measured using a human Th1/Th2 magnetic luminex performance assay 11-plex fixed panel (R&D Systems) according to the manufacturer’s instructions.

### Distribution and Efficacy of YYB-103 Chimeric Antigen Receptor T Cells in a Glioblastoma Xenograft Model

To confirm the *in vivo* distribution of YYB-103 CAR T cells in tumor-bearing mice, 2.5 × 10^5^ U87-luc cells were implanted intracranially into the right forebrain of NOD/SCID mice. On day 16 of tumor implantation, mice were infused with 1.5 × 10^7^ YYB-103 CAR T cells *via* an i.v. route. Mice were sacrificed 3 h, 1 day, 7 days, or 21 days after infusion; and genomic DNA was isolated from the brain, liver, lung, and spleen using DNeasy blood and tissue kits (QIAGEN, Hilden, Germany). qPCR was performed using an Applied Biosystems 7500 (Thermo Fisher) and primer/probe (F: 5′-GAGGCCCTAGAAAGCCAAC-3′, R: 5′-CGAAATCCAGTCCTCTGGTG-3′, reporter: FAM-TGCACGGCTCCGCCAGCAGCT-TAMRA) for YYB-103. DNA plasmid encoding YYB-103 was used as a DNA standard. Seven days after administration of YYB-103 CAR T cells, lymphocytes were enriched from mouse brains using Percoll gradient, and flow cytometric analysis was performed after staining lymphocytes with anti-human CD3-APC (eBioscience) and anti-human CD45-PE-Cy7 (eBioscience).

To evaluate the *in vivo* efficacy of YYB-103 CAR T cells, nude or NSG mice received an orthotopic injection of 1 × 10^5^ U87-luc cells. For i.c.v. infusion, 5 × 10^5^ YYB-103 CAR or UnTd T cells were administered into nude mice 15 days after xenograft. Alternatively, for i.v. injection, 1.5 × 10^7^ of YYB-103 CAR or UnTd T cells were infused into NSG mice 4 days after xenograft. Tumor size was routinely monitored by measuring luminescence signal on a VISQUE inVivo Elite after i.v. injection of 1.5 mg of d-luciferin (Promega, P1043).

### Immunohistochemistry of IL13Rα2 in Glioblastoma Tissue

Records from 211 glioma patients who underwent surgical resection between 2008 and 2019 at our institute were retrieved from the electronic medical record. After review of pathology reports, we confirmed 168 patients with WHO grade 3 and 4 gliomas who underwent resection at our institute. Among these, we identified 53 patients for whom preoperative permission for academic use of tissue sample had been acquired, and we included data from these patients in our analysis. Paraffin-embedded tissue blocks meeting these criteria were retrieved from the archives of the Pathology Section of National Cancer Center. Tissue microarray blocks were prepared for handling multiple samples at once. Briefly, suitable areas for tissue retrieval from 2-mm-diameter cores were punched out from the donor block and inserted into a recipient block. Sections (4 µm thick) were cut from the array block for evaluation. An adequate sample was defined as a tumor occupying more than 10% of the core area.

Immunohistochemistry (IHC) staining was performed using an automated immunostainer (Ventana, Tucson, AZ, USA) according to the manufacturer’s instructions. Anti-IL13Rα2 antibody (Cell Signaling, Danvers, MA, USA; 1:500) was tested and used for IL13Ra2 immunoreactivity as a primary antibody.

### Evaluation of IL13Rα2 Expression by H-Score

**Figure 1:**
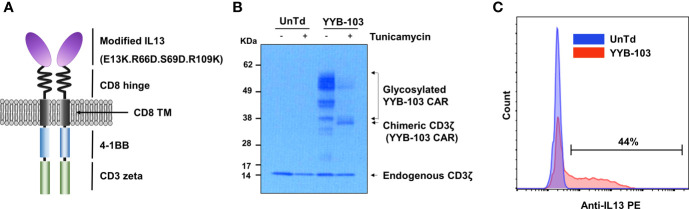
Construction of YYB-103 CAR T cells. **(A)** Schematic diagram of YYB-103 with modified IL13. **(B)** Western blotting analysis of YYB-103. Human peripheral blood mononuclear cells were transduced with YYB-103 retrovirus in the presence or absence of tunicamycin for 24 h, and Western blotting was performed with anti-human CD3ζ antibody and HRP-conjugated secondary antibody. **(C)** Flow cytometric analysis of YYB-103 expression on the cell surface of T cells. CAR, chimeric antigen receptor; HRP, horseradish peroxidase.

H-score was evaluated as previously described (20, 21). The staining intensity of tumor cells was determined as 0 = negative, 1 = faint, 2 = weak, or 3 = moderate to strong. The percentage of positive cells was multiplied by the dominant pattern of staining intensity, producing scores ranging from 0 to 300. For example, an H-score of 270 indicates that 90% of tumor cells showed moderate to strong staining (**Figure 1**).

### Statistical Analysis

Comparison of H-score between groups according to designated variables was performed using Student’s t-tests. Pearson’s correlation coefficients were used to evaluate the correlation between two continuous variables. We considered a *p*-value < 0.05 to indicate statistical significance. All statistical analyses were performed using SPPS (version 18.0, Chicago, IL).

## Results

### Generation of IL13Rα2-Specific YYB-103 Chimeric Antigen Receptor T Cells

Previous studies report that the amino acid substitution of IL13 reduces binding affinity to IL13Rα1 in normal cells (22–24). YYB-103 contains a modified IL13 with substituted amino acids as an antigen-binding domain to increase its selectivity for the target protein IL13Rα2 while reducing its binding affinity for IL13Rα1 expressed on normal cells. Modifications of IL13 in YYB-103 include substitution of the 13th, 66th, 69th, and 109th amino acid sequences in WT-IL13 with E13K, R66D, S69D, and R109K. YYB-103 consists of an Ig heavy chain signal peptide, optimized antigen-binding domain, human CD8 hinge, human CD8 transmembrane domain, 4-1BB intracellular signaling domain, and CD3ζ-signaling domain (**Figure 1A**).

To determine whether YYB-103 was normally expressed in human T cells, we transduced YYP-103 into human T cells and performed Western blotting with anti-CD3ζ monoclonal antibody (mAb) using cell lysates from cultured YYB-103 CAR and UnTd T cells. In the UnTd T cells, only endogenous CD3 zeta proteins were found, while various sizes of proteins were detected with anti-CD3ζ mAb in YYB-103 CAR T cells (**Figure 1B**). A homogenous band of ~35 kDa was detected with anti-CD3ζ mAb following treatment of tunicamycin, an inhibitor of N-linked glycosylation in contrast to multiple >35-kDa bands without the inhibitor (**Figure 1B**), indicating that YYB-103 proteins were actively glycosylated in human T cells. Flow cytometric analysis with anti-human IL13 PE antibody further indicated that YYB-103 was stably expressed on the surface of human T cells (**Figure 1C**). These results indicate that YYB-103 was translated, post-translationally modified, and properly expressed on the surface of human T cells.

### YYB-103 Chimeric Antigen Receptor T Cells Preferentially Recognize IL13Rα2^+^ Tumor Cells

Surface plasmon resonance analysis was conducted to compare the binding affinity of WT and modified IL13 in YYB-103 to human IL13Rα1 and IL13Rα2. The equilibrium dissociation constant (K_D_) for human IL13Rα1 was 19.73 nM for WT-IL13 but was unmeasurable for modified IL13 due to nearly absent binding. We also compared the binding affinity of several IL13 muteins, including previously reported IL13 muteins (E13Y) (13, 17), to IL13Rα1 and confirmed that modified IL13 (E13K.R66D.S69D.R109K) in YYB-103 showed little binding to IL13Rα1 as compared with other IL13 muteins under the same conditions (**Supplementary Figure 1A**). WT-IL13 bound to IL13Rα2 with a K_D_ of 0.5853 nM, whereas YYB-103 IL13 bound to IL13Rα2 with a K_D_ of 7.401 nM, indicating that YYB-103 had lower binding affinity for IL13Rα2 than WT-IL13 (**Table 1** and **Supplementary Figure 1B**).

**Table 1:** Measurement of affinity of WT-IL13 and YYB-103 IL13 for human IL13Rα1 and IL13Rα2.

Ligand	Analyte	K_a_ (M^−1^ S^−1^)	Kd (S^−1^)	K_D_ (nM)
Human IL13Rα1	WT-IL13^€^	3.202e+4	6.319e−4	19.73
	YYB103 IL13^¥^	N/D	N/D	N/D
Human IL13Rα2	WT-IL13^€^	3.031e+4	1.774e−5	0.5853
	YYB103 IL13^¥^	1.669e+3	1.257e−5	7.401

IL13, interleukin-13; IL13Rα, IL13 receptor alpha; K_a_, association constant; Kd, dissociation constant; K_D_, equilibrium dissociation constant; N/D, not detected; WT, wild type.

^￥^Modified IL13 fused with human Fc.

^€^WT-IL13 fused with human Fc.

**Figure 2:**
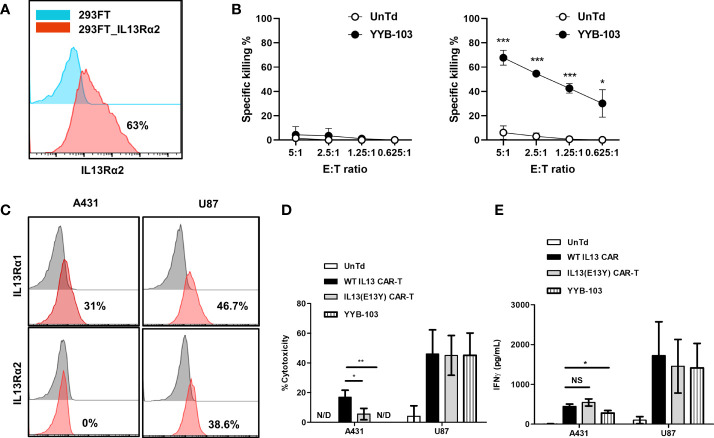
Modified IL13 in YYB-103 CAR T cells preferentially binds to IL13Rα2 and not IL13Rα1 on tumor cells. **(A)** Flow cytometry confirmation of IL13Rα2 expression in naïve (blue) and IL13Rα2-transfected (red) 293FT cells. **(B)** *In vitro* cytotoxicity assay was performed by co-culturing CAR T cells with naive (left) and IL13Rα2-transfected (right) 293FT cells for 18 h. **(C)** Flow cytometry evaluation of IL13Rα1/IL13Rα2 expression revealed IL13Rα1^+^/IL13Rα2^−^ for A431 cells and IL13Rα1^+^/IL13Rα2^+^ for U87 cells. **(D)** *In vitro* cytotoxicity assay was performed by co-culturing different IL13 muteins CAR T cells with A431 (IL13Rα1^+^/IL13Rα2^−^) or U87 (IL13Rα1^+^/IL13Rα2^+^) cells for 18 h. **(E)** IFN-γ production of CAR T cells was measured in culture supernatant after co-culture with A431 or U87 cells. Statistical significance was determined by using the unpaired two-tailed Student’s t-test for comparisons between two samples. *p < 0.05, **p < 0.01, ***p < 0.005). E:T ratio, effector-to-target cell ratio; CAR, chimeric antigen receptor. NS, not significant; N/D, not detected.

To confirm the cytotoxicity of YYB-103 CAR T cells toward IL13Rα2^+^ cells, IL13Rα2 was stably expressed on 293FT human embryonic kidney cells (**Figure 2A**), which were used as target cells. YYB-103 CAR T cells were co-cultured with parental 293FT or 293FT-IL13Rα2 cells with various E:T ratios, which showed that YYB-103 CAR T cells did not induce cytotoxicity toward parental 293FT cells that did not express IL13Rα2 but induced strong cytotoxicity toward 293FT-IL13Rα2 cells (**Figure 2B**). Using IL13Rα1^+^/IL13Rα2^−^ A431 and IL13Rα1^+^/IL13Rα2^+^ U87 cells (**Figure 2C**), we further confirmed whether YYB-103 CAR T cells selectively induced cytotoxicity toward target cells expressing IL13Rα2 but not IL13Rα1. CAR T cells expressing YYB-103 and WT-IL13 and UnTd T cells were co-cultured with IL13Rα1^+^/IL13Rα2^−^ A431 or IL13Rα1^+^/IL13Rα2^+^ U87 cells. Cytotoxicity assay showed that UnTd T cells rarely induced cytotoxicity toward A431 or U87 cells, WT-IL13 CAR T cells induced cytotoxicity toward both A431 and U87 cells, and, as expected, YYB-103 CAR T cells selectively killed IL13Rα2^+^ U87 but not IL13Rα2^−^ A431 cells (**Figure 2D**). Furthermore, we compared cytotoxicity between YYB-103 CAR T cells and previously described CAR T cells with IL13 (E13Y) modification (13). As the previous IL13 (E13Y) showed mild toxicity toward A431 cells, it was verified that YYB-103 CAR T cells have superior selective cytotoxicity to the IL13 (E13Y) toward IL13Rα2^−^ cells (**Figure 2D**).

We achieved similar results for IFN-γ production, showing that the IFN-γ expression of YYB-103 CAR T cells was significantly reduced when they were co-cultured with IL13Rα1^+^/IL13Rα2^−^ A431 cells but not IL13Rα2^+^ U87 cells. However, E13Y CAR T cells did not show lower IFN-γ production when co-cultured with IL13Rα1^+^/IL13Rα2^−^ A431 cells compared with WT-IL13 CAR T-cells (**Figure 2E**). Other cytokines, including GM-CSF, IL4, and IL2, were also produced by YYB-103 CAR T cells upon exposure to IL13Rα2-expressing cells such as 293FT-IL13Rα2 and IL13Rα2^+^ U87 cells, while those were not detectable in both IL13Rα2^−^ 293FT and A431 cells (**Supplementary Figure 2**).

These results indicate that WT-IL13 bound to both IL13Rα1 and IL13Rα2, whereas the modified IL13 in YYB-103 selectively bound to IL13Rα2 but not IL13Rα1, demonstrating improved selectivity compared with previously developed CAR T cells with IL13 (E13Y) modification. Thus, YYB-103 CAR T cells may show improved preferential cytotoxic effects toward IL13Rα2-expressing tumor cells.

### YYB-103 Chimeric Antigen Receptor T Cells Suppress Tumor Growth in an Orthotopic Glioblastoma Model

Although YYB-103 CAR T cells successfully killed IL13Rα2^+^ U87 cells *in vitro*, it was not clear whether they could kill GBM in the brain by penetrating the blood–brain barrier (BBB), which restricts the passage of peripheral immune cells.

**Figure 3:**
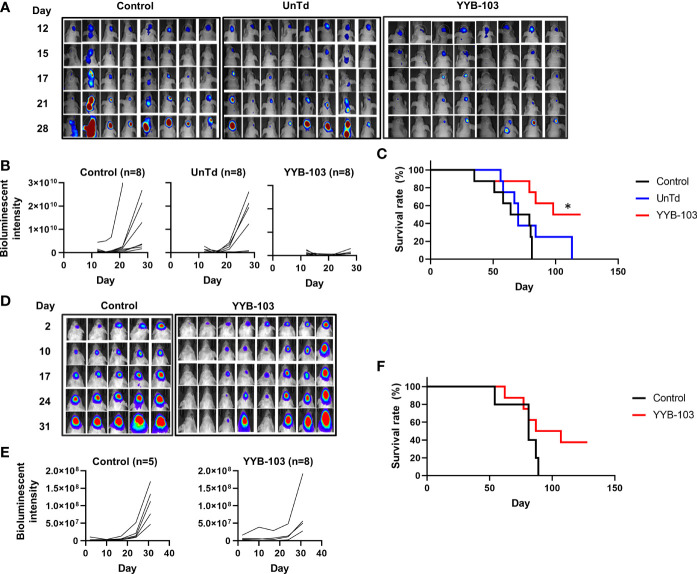
*In vivo* antitumor activity of YYB-103 CAR T cells. (*Top row*) Nude mice were orthotopically injected with U87 human glioma cells expressing luciferase (U87-luc) and further received PBS, UnTd T cells, or YYB-103 CAR T cells *via* an i.c.v. route. Survival and tumor growth were routinely monitored. **(A)** Optical imaging of U87-luc cells in nude mice were obtained using VISQUE *in vivo* Elite on the indicated days. **(B)** Bioluminescence intensities were calculated from the images. **(C)** Survival rate of each group of mice. Log-rank Mantel–Cox test was performed, *p < 0.05. (*Bottom row*) Control (PBS) and YYB-103 CAR T cells were i.v. injected into NSG mice that were orthotopically injected with U87-luc. Survival and tumor growth were routinely monitored. **(D)** Optical imaging of U87-luc cells in NSG mice on the indicated days. **(E)** Bioluminescence intensities were calculated from the images. **(F)** Survival rate of each group of mice. CAR, chimeric antigen receptor; PBS, phosphate-buffered saline; UnTd, untransduced; i.c.v., intracerebroventricular; i.v., intravenous.

First, we examined whether direct administration of YYB-103 CAR T cells into the brain *via* the i.c.v. route would eradicate IL13Rα2^+^ U87 cells. IL13Rα2^+^ U87 cells were implanted into nude mouse brains, and YYB-103 CAR or UnTd T cells were injected i.c.v. into mice 15 days after xenograft of U87 cells following confirmation of tumor growth by bioluminescence (**Figure 3A**). Consistent with previous reports (25, 26), YYB-103 CAR T cells suppressed the growth of implanted tumor cells in the brain when 5 × 10^5^ cells were i.c.v. injected, whereas UnTd T cells minimally affected tumor growth (**Figures 3A, B**). Consequently, the survival rate of U87-implanted mice was significantly improved by YYB-103 CAR T cells (*p* = 0.0058) but not by UnTd T cells, resulting in the survival of approximately half of mice until the end of the study (**Figure 3C**).

Next, 1 × 10^7^ YYB-103 CAR T cells were injected i.v. into mice 4 days after orthotopic injection of U87 cells. Tumor growth rate measured by bioluminescence was significantly reduced by i.v. injection of YYB-103 CAR T cells compared with control (*p* = 0.048, **Figures 3D, E**). The median survival time of vehicle-treated mice (81 days) was improved by injection of YYB-103 CAR T cells (97 days), although this difference failed to reach statistical significance due to the small number of treated mice (*p* = 0.104, log rank test) (**Figure 3F**). These results suggest that YYB-103 CAR T cells have the potential to kill IL13Rα2^+^ U87 cells, possibly by infiltrating the inflamed region of the brain by crossing the BBB.

### *In Vivo* Distribution of Intravenous Injected YYB-103 Chimeric Antigen Receptor T Cells

**Figure 4:**
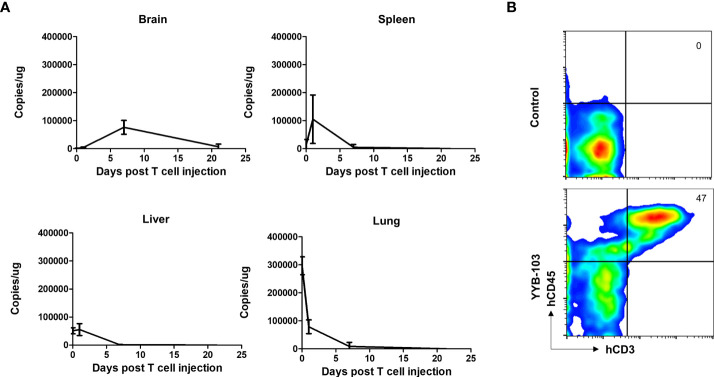
Biodistribution of i.v. injected YYB-103 CAR T cells in a mouse tumor model. NOD/SCID mice were orthotopically injected with U87-luc and further administered YYB-103 CAR T cells 16 days after tumor implantation. Brain, spleen, liver, and lung tissues were collected at 3 h (*n* = 5), 1 day (*n* = 5), 7 days (*n* = 4), or 21 days (*n* = 2) after the CAR T i.v. injection. **(A)** Quantity of YYB-103 CAR T cells measured by qPCR of genomic DNA for the YYB-103 transgene in each organ on the indicated days. **(B)** Representative flow cytometry plots of human T cells infiltrated into mouse brain. Seven days after administration of YYB-103, lymphocytes were isolated from mouse brains using Percoll, and fluorescence-activated cell sorting analysis was performed using human CD3-APC and CD45 PE-Cy7. i.v., intravenous; CAR, chimeric antigen receptor.

As i.v. injection of YYB-103 CAR T cells successfully suppressed tumor growth in the brain, we sought to confirm whether these cells infiltrated into the brain across the BBB. YYB-103 CAR T cells were i.v. injected into NOD/SCID mice 16 days after orthotopic injection of U87 tumor cells. We collected tissue samples at various time points and quantified genomic YYB-103 DNA in the brain, liver, lung, and spleen using qPCR analysis with a primer set for the YYB-103 transgene. Three hours after infusion, YYB-103 CAR T cells were maximally detected in the lung and rarely detected in other organs (**Figure 4A**). YYB-103 peaked in the spleen and liver on day 1 and became undetectable in the spleen, liver, and lung 7 days after infusion (**Figure 4A**). By contrast, YYB-103 CAR T cells gradually increased in the brain, peaked at day 7, and lasted until day 20 (**Figure 4A**). Flow cytometry of brain tissue also readily detected human CD3^+^ T cells in the brain of mice that received YYB-103 CAR T cells (**Figure 4B**).

These results suggest that YYB-103 CAR T cells infused *via* the i.v. route initially accumulated in the lung and then migrated to secondary lymphoid organs and liver, with some infiltrating into the inflamed region of the brain across the BBB.

### IL13Rα2 Expression in Normal and Glioma Tissue

To ensure the safety of YYB-103 CAR T cells, IL13Rα2 should be expressed on tumor cells but not on cells from normal tissue. We examined the possible expression of IL13Rα2 by IHC analysis using the Normal Human Tissue Microarray as described in the *Materials and Methods* section and confirmed that most organs, except the cell membrane of the testes, showed no IL13Rα2 expression (**Supplementary Figure 3**).

To analyze IL13Rα2 expression in glioma, 53 MG samples (summarized in **Table 2**, five non-tumor brain samples summarized in **Supplementary Table 1**) were collected and used for IL13Rα2 IHC. Thirty-two patients were male, and 21 patients were female. Median patient age was 61.3 years (range, 14–90 years). Histologic diagnoses were WHO grade 4 GBM for 46 samples, grade 3 anaplastic oligodendroglioma for four samples, and grade 3 anaplastic astrocytoma for three samples. Based on previous histologic diagnosis, five cases had transformed to GBM (secondary) from anaplastic astrocytoma (*n* = 2), diffuse astrocytoma (*n* = 2), and oligodendroglioma (*n* = 1); and the other 48 cases were *de novo* MG. In addition to these five secondary GBM samples, five other samples were recurrent cases.

**Table 2:** H-score for IL13Rα2 in malignant glioma samples according to clinical characteristics (*n* = 53).

Characteristics	Number of samples	H-score,mean (SD)	*p-*Value
Age			0.70
<65 years	32	61.3 (85.5)	
≥65 years	21	51.7 (91.4)	
Gender			0.59
Female	20	49.1 (76.9)	
Male	33	62.5 (93.6)	
WHO grade			<0.001
Grade 3	7	1.6 (3.7)	
Grade 4	46	66.0 (90.6)	
Origin			0.66
*De novo*	48	59.1 (88.6)	
Secondary	5	40.8 (78.3)	
Primary *vs.* recurrent			0.50
Primary	4	61.4 (89.8)	
Recurrent	10	40.4 (76.5)	
Multiplicity			0.86
Single	11	56.4 (86.4)	
Multiple	42	61.6 (94.4)	

IL13Rα2, interleukin-13 receptor alpha 2.

**Figure 5:**
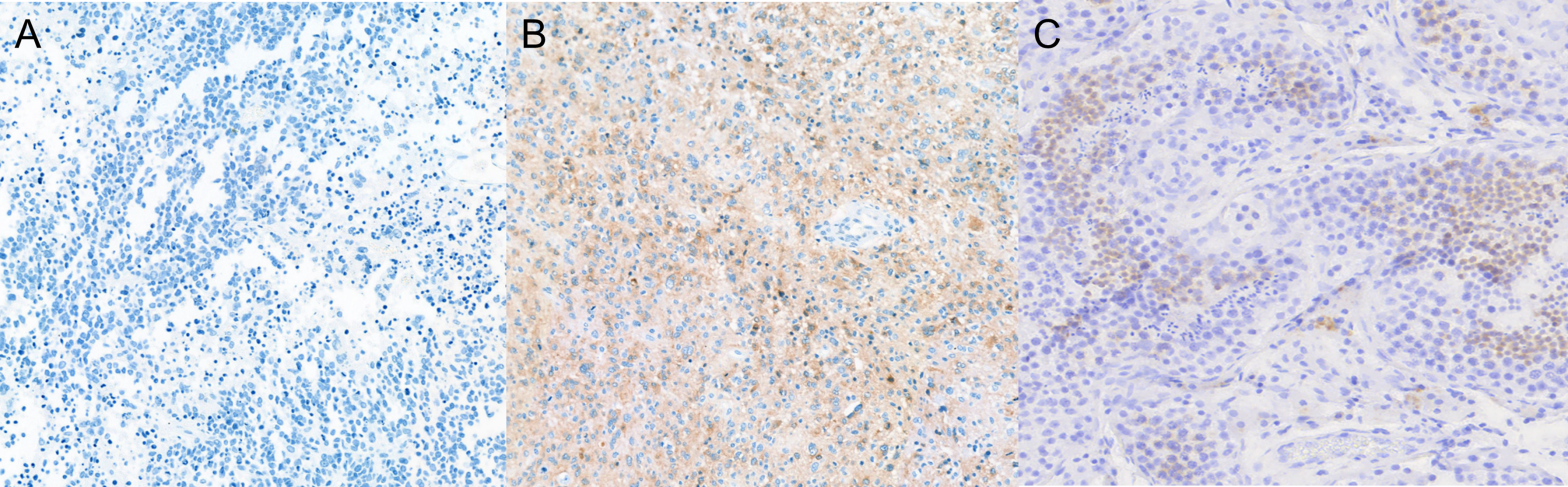
Expression of IL13Rα2 in glioma and testes tissues. Glioma and testes tissues were stained with anti-IL13Rα2 antibody and HRP-conjugated secondary antibody. **(A)** H-score of 0 (0 intensity × 0% proportion). **(B)** H-score of 270 (3 intensity × 90% proportion). **(C)** Testes as a positive control (×100 magnification). HRP, horseradish peroxidase.

**Figure 6:**
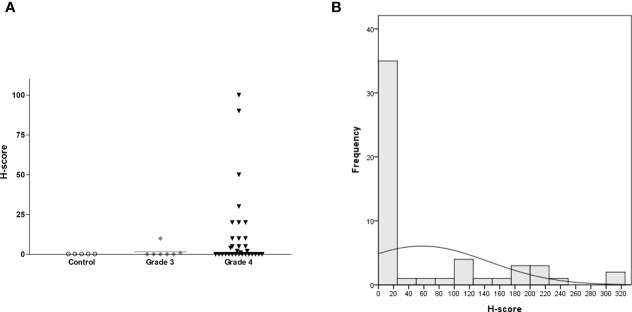
H-score distribution for IL13Rα2 IHC staining according to glioma grade with non-tumorous brain tissue as a negative control shown as a **(A)** scatterplot (*n* = 58, horizontal bar representing median value) and **(B)** histogram (*n* = 53 for MG). IHC, immunohistochemistry; MG, malignant glioma.

H-score was calculated by multiplying IHC staining intensity by the proportion of positive cells in the tumor section (**Supplementary Table 1** and **Figure 5**). All five controls were negative for IL13Rα2 expression (**Figure 6A**). The intensity of IL13Rα2 staining was 0 for 20 MG samples (38%), 1 for nine samples (17%), 2 for 20 samples (38%), and 3 for four samples (8%). The proportion of positive cells ranged widely from 0% to 100%, with a median of 5.0% and mean of 26.7% (SD, 37.2). IL13Rα2 staining intensity and proportion of positive cells were positively correlated (*r* = 0.698, *p* < 0.01; **Supplementary Figure 4**). The H-score distribution (range, 0–300) was positively skewed (skewness = 1.21), with a median of 5 and a mean of 57.5 (SD, 87.2; **Figure 6B**). Twenty samples (38%) showed no staining for IL13Rα2 and thus had an H-score of 0, and 16 samples (30%) had an H-score of ≥100, including two samples with an H-score of 300.

### IL13Rα2 H-Score According to Clinical Variables and Molecular Markers

We next evaluated H-score for IL13Rα2 in MG samples according to clinical variables. H-score did not differ according to age (<65 *vs*. ≥65 years), gender, MG origin (*de novo vs*. secondary), or treatment history (primary *vs*. recurrent) (**Table 2**). However, H-score was related to histological grade. Grade 4 GBM samples showed strong IL13Rα2 expression with a mean H-score of 66.0 (SD, 90.6), which was significantly higher than that of grade 3 MGs (1.6, *p* < 0.001). The MRI finding of multiple lesions was not significantly associated with H-score.

Molecular markers of MG were examined at the discretion of the pathologist who helped make a final histologic diagnosis. IHC evaluation was performed for P53 (*n* = 37), isocitrate dehydrogenase-1 (IDH-1) mutation (*n* = 37), epidermal growth factor receptor (EGFR; *n* = 25), synaptophysin (*n* = 15), and vimentin (*n* = 8). *O* ^6^-Methyl guanine methyl-transferase (MGMT) methylation status was examined by methylation-specific (nested) PCR (*n* = 29). As some of these markers are exclusively expressed according to histologic grade and diagnosis, we analyzed H-score for IL13Rα2 according to molecular marker expression only in GBM samples (**Table 3**). The IDH-1 WT group had a significantly higher H-score than the IDH-1 mutant group (mean, 72.7 *vs*. 6.7, *p* = 0.003). Also, MGMT-unmethylated GBMs had a significantly higher H-score than MGMT-methylated GBMs (mean, 78.0 *vs*. 5.0, *p* = 0.002). These results suggest that patients with unfavorable molecular markers for GBM standard treatment could benefit from YYB-103 CAR T-cell therapy.

**Table 3:** H-score for IL13Rα2 in malignant glioma samples according to molecular markers (*n* = 46).

Molecular markers	Number of samples	H-score,mean (SD)	*p-*Value
P53			0.33
(+)	21	72.0 (104.9)	
(−)	11	38.6 (68.3)	
EGFR			0.57
(+)	19	24.0 (56.6)	
(−)	3	4.67 (5.03)	
IDH-1			0.003
Mutant	3	6.7 (11.5)	
Wild type	27	72.7 (99.1)	
Synaptophysin			0.45
(+)	4	48.8 (87.6)	
(−)	9	101.8 (122.1)	
MGMT			0.002
Methylated	3	5.0 (5.0)	
Unmethylated	23	78.0 (99.6)	

EGFR, epidermal growth factor receptor; IDH-1, isocitrate dehydrogenase-1; IL13Rα2, interleukin-13 receptor alpha 2; MGMT, O^6^-methyl guanine methyl-transferase.

As we plan to set an H-score >0 an as IHC screening criterion for a future YYB-103 CAR T-cell clinical trial, we examined the distribution of each clinical variable to evaluate potential risk factors for screening failure. The clinical variables of age (<65 *vs*. ≥65 years), gender, MG origin (*de novo vs*. secondary), treatment history (primary *vs*. recurrent), and presence of multiple lesions were not associated with whether H-score of 0 or >0 (**Supplementary Table 2**). WHO grade tended to be associated with whether H-score was 0 or >0, but this did not reach statistical significance (*p* = 0.09). Based on these results, we expect that none of these clinical variables would serve as additional screening exclusion criteria.

## Discussion

CAR T-cell adoptive immunity is an alternative method of overcoming immune tolerance in the autologous setting. However, CAR T-cell immunotherapy for solid tumors remains challenging, largely due to the lack of appropriate surface antigens whose expression is confined to malignant tissue to avoid “on-target” toxicity from “off-tumor” expression (27). We found that YYB-103 CAR T cells showed effective cytotoxicity toward tumor cells expressing IL13Rα2. Animal experiments involving orthotopic implantation of U87 MG show that i.v. administration of YYB-103 CAR T cells successfully inhibits cancer cell growth and extends the survival of mice through migration of YYB-103 CAR T cells to the brain. The present preclinical studies of YYB-103 CAR T cells demonstrate that they can be safely applied to treat MG by discriminating normal and malignant cells in the brain.

### Benefits and Drawbacks of Intravenous Administration of Chimeric Antigen Receptor T Cells

Factors limiting i.v. administration of CAR T cells include on-/off-target toxicity and possibly lower effectiveness than local administration. IL13Rα1 is expressed in most normal organs, including the brain; and such ubiquitous expression can interfere with i.v. administration of CAR T cells that use IL13 as an antigen-binding domain. However, as the modified IL13 has low affinity for IL13Rα1, no significant side effects were observed after i.v. administration in immunotoxin clinical trials involving conventional IL13 (14). Moreover, no specific off-target toxicity was identified in the City of Hope-led clinical trials despite local administration to the brain (13). Our findings confirm that modified IL13 in YYB-103 lowers affinity for IL13Rα1 compared with conventional IL13, resulting in the absence of cytotoxicity toward cells that express IL13Rα1. Our data show that i.v. administration of YYB-103 CAR T cells did not show any toxicities from human IL13 cross-reaction with mouse IL13Rα1. In particular, we did not observe any signs of respiratory difficulty in the mice in spite of the relatively rapid distribution of YYB-103 CAR T cells to the lungs. Based on these results, we suggest that the possibility of off-target toxicity toward IL13Rα1 *via* i.v. administration is very low. However, as our data show that WT-IL13 CAR-T and IL13(E13Y) CAR-T revealed low cytotoxicity for IL13Rα1-positive cells despite very low but retained affinity for IL13Rα1, a possible unexpected off-target toxicity using modified IL13 in YYB-103 still needs to be evaluated in future clinical trials.

Although IL13Rα2, which has high affinity for WT-IL13, has been observed in other organs, our results confirm that its expression is confined to the testes. Despite that high expression of IL13Rα2 is highly expressed in the testes, no side effects were reported in clinical trials using IL13 toxins (14). In addition, if on-target toxicity toward IL13Rα2 in the testes occurs, the toxicity is assumed to be clinically controllable, as it is expected to be limited to fertility.

Another challenge of i.v. administration for central nervous system (CNS) tumors is the possibility of a lower degree of effectiveness than local administration due to a paucity of tumor-infiltrating lymphocytes compared with systemic cancer, as the brain is known to be an “immunoprivileged site.” Therefore, local administration of CAR T cells is generally preferred over i.v. administration because i.v. infused drug may neither cross the BBB nor be able to achieve an effective concentration in the brain. However, in a previous clinical trial of i.v. infused EGFRvIII-directed CAR T cells, the trafficking of CAR T cells to brain tumors was verified directly by IHC staining of the posttreatment tumor tissues from a palliative salvage operation (28). Also, in our mouse orthotopic xenograft model, YYB-103 CAR T cells gradually increased in the brain and peaked at day 7, which confirms that systematically delivered CAR-T can be migrated to the tumor site within the CNS. In brain tumor environments, BBB is generally disrupted by a tumor (29, 30), and macromolecules such as drugs and mAbs have been proven to penetrate the BBB (31). Thereby, YYB-103 CAR T cells might migrate across the BBB by not only passively crossing the leaky BBB but may also be attracted partly by chemokine–chemokine receptor interaction (32), the engagement of adhesion molecules (30), and activation status by tonic signaling (33, 34). However, further studies are required to determine the exact mechanisms.

Although intratumoral administration may theoretically have a greater anticancer effect than i.v. administration in view of direct delivery, intratumoral administration has limited effect to multifocally spread tumors, which was the nature of gliomas. In a previous clinical trial of i.c.v. infused IL13Rα2-targeted CAR T cells (19) and i.v. infused Her2-specific CAR-modified virus-specific T cells (VSTs) (25), efficacy of CAR T cells to multifocal brain tumors was observed. Therefore, i.v. or i.c.v administration, which may allow CAR T cells to be delivered to the whole brain, likely has more greater anticancer efficacy against multifocal gliomas than intratumoral administration as long as a sufficient number of CAR T cells migrate.

### IL13Rα2 Expression According to Different Histological and Clinical Factors of Malignant Glioma

Although the level of IL13Rα2 expression according to glioma grade has been investigated using only a small number of samples, researchers report increased IL13Rα2 expression in proportion to higher glioma grade. Specifically, in a previous study of pediatric primary brain tumors by Kawakami et al., 100% (11 of 11) of high-grade astrocytomas and 79% (26 of 33) of low-grade astrocytomas expressed IL13Rα2 (7). Although based on tentative classification of expression level, the authors found that IL13Rα2 is weakly expressed in low-grade gliomas other than MG. Brown et al. also examined IL13Rα2 expression according to glioma grade and histological subtype (35). The authors found that GBM exhibited 3.5-fold higher expression than all other grades, but there were no significant differences among grade 2/3 astrocytoma, oligoastrocytoma, and oligodendroglioma. Although the number of grade 3 MG samples was small in our study, the H-score of IL13Rα2 expression was significantly lower in grade 3 MG compared with GBM (mean, 1.6 *vs*. 66.0).

The association of IL13Rα2 expression with molecular subtype or other molecular markers has not been extensively studied. Among four molecular subtypes of GBM (36), Brown et al. found that IL13Rα2 expression was positively correlated with the mesenchymal subtype and negatively correlated with the proneural subtype using a publicly available database and Affymetrix microarray template (35). IL13Rα2 is a binding partner of the glycosyl hydrolase family 18 chitinase 3-like-1 (Chi3li, YKL-40 in humans), which is a marker of the mesenchymal subtype of GBM (37). Chi3li augments transforming growth factor-β1 (TGF-β1) production *via* an IL13Rα2-dependent mechanism (38). However, the detailed regulatory mechanism of co-expression or interaction between YKL-40 and IL13Rα2 remains unknown.

Steroid and radiation therapy for GBM are known to cause immunosuppression (39). However, in the present study, samples from MG patients who were previously treated showed similar IL13Rα2 expression as those from patients with naïve primary MG, although the dose, modality, and time of treatment before biopsy varied substantially across patients.

### Correlation Between Target Antigen Expression and Efficacy of Chimeric Antigen Receptor T Cells

The present study shows that the expression of IL13Rα2 varies among MG samples, with 38% of cases showing no staining. Many *in vitro* studies report a correlation between target antigen expression and efficacy of CAR T cells, with antigen density having a particularly large impact on the efficacy of CAR T cells (40–42). However, contrary to these *in vitro* studies, CD19 CAR T-cell clinical trials do not indicate a correlation between the degree of target antigen expression and clinical response in lymphoma (43) or multiple myeloma (44). In this context, it is not easy to set a threshold for target antigen expression in clinical practice based on animal studies, and it is possible that applying a high threshold based on non-clinical data would exclude patients who can be treated. In previous clinical trials of dendritic cell vaccination, pseudo-toxin, or CAR T cells, the cytotoxic effects of IL13/IL13Rα2-based treatments were not in proportion to the level of IL13Rα2 expression in tumors (11, 45, 46). This can be explained by the complex mechanism of immunotherapy involving antigen heterogeneity, successful expansion of CAR T cells, and activation of the “cancer immunity cycle” (47).

Previous clinical trials of CAR T cells for solid cancers, including GBM, set a threshold for target expression as low as possible (13, 25, 28, 48). Clinical trials of IL13Rα2-targeted (13) and Her2-targeted (25) CAR T cells for GBM applied a proportion of positive cells ≥20% and an IHC staining intensity of ≥1. Another clinical trial of IL13Rα2-targeted CAR T cells for recurrent GBM (NCT04003649) applied an H-score of ≥50. In the Her2-targeted CAR T-cell trial, patients with grade 1 (1%–25%) or higher were included by IHC (25). In the clinical trial for EGFRvIII-targeted CAR T cells, which employed next-generation sequencing, patients with EGFRvIII ratios (EGFRvIII reads/[WT EGFR reads + EGFRvIII reads]) > 6% were enrolled (28). To date, there are no sufficient data available from these clinical trials to determine the correlation between target antigen expression and efficacy; thus, more data are required to infer an appropriate threshold for efficacy.

## Conclusion

Based on the results of this preclinical study, we expect that i.v. administration of IL13Rα2-targeted YYB-103 CAR T cells will have anticancer effects in MG patients. The high H-score for IL13Rα2 in GBM, especially in conjunction with the poor prognostic markers of WT IDH-1 WT and unmethylated MGMT, encourage us to move forward in planning a clinical trial for recurrent MG.

## Data Availability Statement

The raw data supporting the conclusions of this article will be made available by the authors, without undue reservation.

## Ethics Statement

The studies involving human participants were reviewed and approved by the Institutional Review Board (IRB) of the Korea National Institute for Bioethics Policy (P1-201510-31-005). The patients/participants provided their written informed consent to participate in this study. The animal study was reviewed and approved by CellabMED Institutional Animal Care and Use Committee (CLMIACUC; AEC-20191112-0002).

## Author Contributions

KK: conceived and designed the experiments, performed the animal experiments (**Figure 4**), and wrote the paper. H-SG: conceived and designed the experiments, performed the experiments (**Figures 5** and **6**), and wrote the paper. NH: performed the IHC experiments (**Figure 5**) and wrote the paper. EH: conceived and designed the experiments and performed the IHC experiments (**Figure 5**). BC: conceived and designed the experiments and wrote the paper. SL: performed the IHC and animal experiments (**Figure 3** and **Supplementary Figure 3**). SC: performed the *in vitro* experiments (**Figure 2** and **Supplementary Figure 1**). J-HP: manufactured CAR-T and performed the *in vitro* experiments (**Supplementary Figure 2**). J-HS: manufactured CAR-T and performed the *in vitro* experiments (**Figure 2** and **Supplementary Figure 2**). YJ: performed the Western blotting experiments (**Figure 1**). HC: conceived and designed the experiments. S-JL: conceived and designed the experiments. YL: performed the IHC experiments (**Supplementary Figure 3**). KN: conceived and designed the experiments. S-WS: conceived and designed the experiments. All authors contributed to the article and approved the submitted version.

## Funding

This research was financially supported by National Cancer Center Graduate School of Cancer Science and Policy Research and Development Business Foundation (202000460001) and the Korea Ministry of SMEs and Startups under the “Regional Specialized Industry Development Program” (non-R&D, R0005140) supervised by the Korea Institute for Advancement of Technology.

## Conflict of Interest

KK, SL, SC, J-HP, JS, YJ, HC, and S-JL are employed by *CellabMED*, and S-WS is a shareholder of *CellabMED*.

The remaining authors declare that the research was conducted in the absence of any commercial or financial relationships that could be construed as a potential conflict of interest.

## Publisher’s Note

All claims expressed in this article are solely those of the authors and do not necessarily represent those of their affiliated organizations, or those of the publisher, the editors and the reviewers. Any product that may be evaluated in this article, or claim that may be made by its manufacturer, is not guaranteed or endorsed by the publisher.
