# Supplementary material for: Chimeric Antigen Receptor T Cells With Modified Interleukin-13 Preferentially Recognize IL13Rα2 and Suppress Malignant Glioma: A Preclinical Study
Source: Front Immunol. 2021 Nov 8;12:715000. doi: 10.3389/fimmu.2021.715000 (PMC8606595; doi:10.3389/fimmu.2021.715000)
Supplement: Supplementary file 1 [file DataSheet_1.docx]

**A**

**
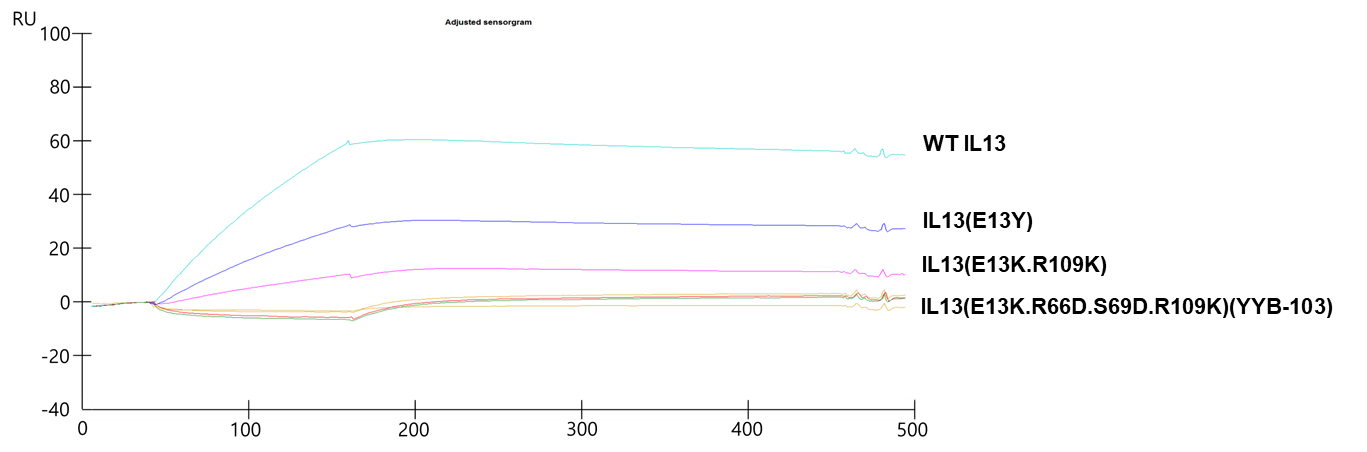
**

**B**
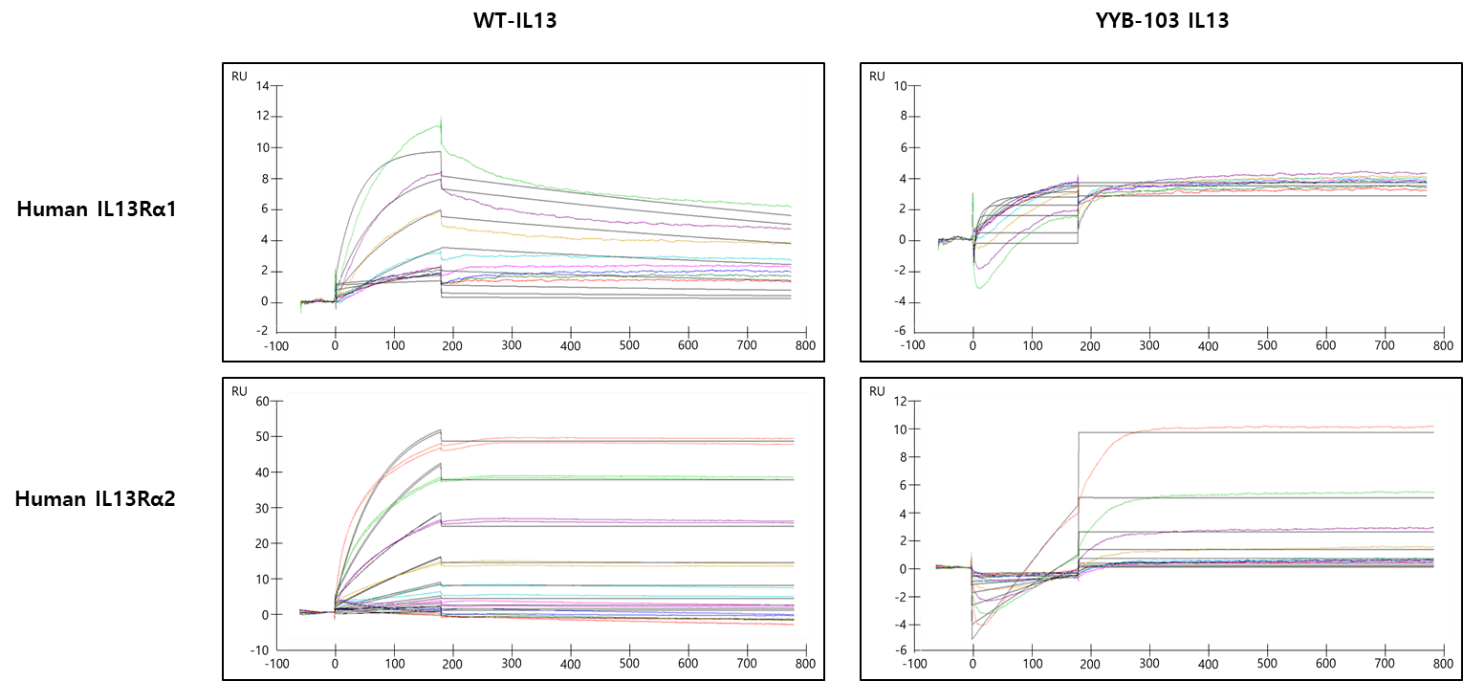


**Supplementary Figure 1.** Binding affinity of wild type and modified IL13 to IL13Rα1 to evaluate selective binding to IL13Rα2. (A) Screening of modified IL13 (B) Binding affinity of WT-IL13 and YYB-103 IL13 for human IL13Rα1 and IL13Rα2.

**Supplementary Figure 2**. Cytokine secretion of UnTd and YYB-103 CAR T cells upon contact with different target cells: Hek293FT, Hek293FT_IL13Rα2, A431, and U87. **p* < 0.05 ****p* < 0.005).


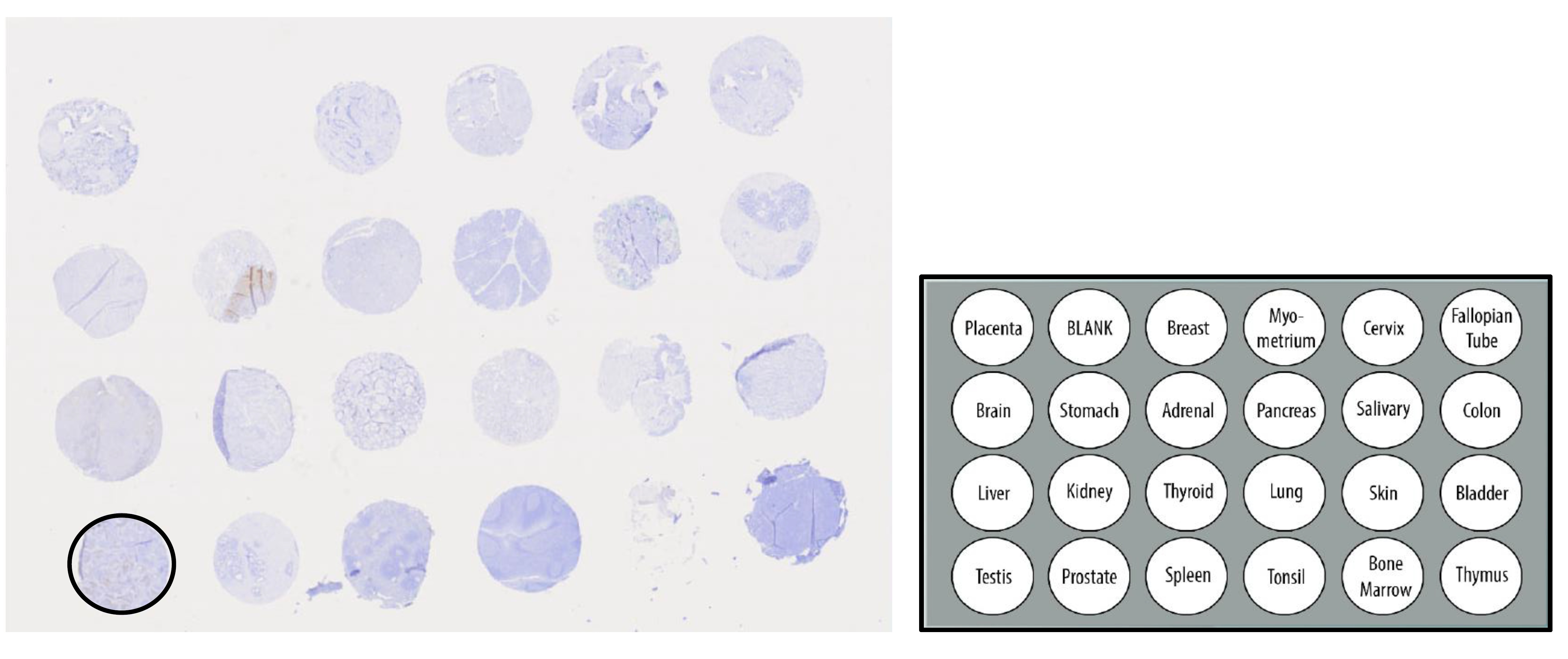


**Supplementary Figure 3**. IHC of IL13Rα2 with 23 Core Normal Human Tissue Microarray.


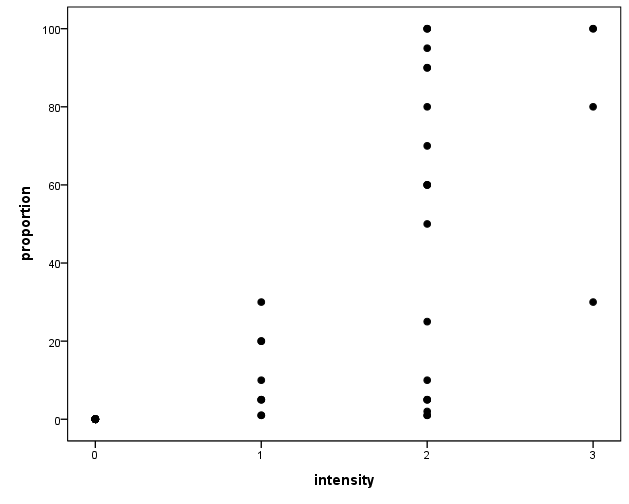


**Supplementary Figure 4.** Scatterplot for correlation between the intensity and proportion of cells positive for IL13Rα2 IHC staining (*r* = 0.713, *p* < 0.001).
