# Supplementary material for: Chimeric Antigen Receptor T Cells With Modified Interleukin-13 Preferentially Recognize IL13Rα2 and Suppress Malignant Glioma: A Preclinical Study
Source: Front Immunol. 2021 Nov 8;12:715000. doi: 10.3389/fimmu.2021.715000 (PMC8606595; doi:10.3389/fimmu.2021.715000)
Supplement: Supplementary file 3 [file Table_2.docx]

**Supplementary Table 2.** Distribution of clinical characteristics according to IL-13Rα2 expression in malignant glioma (*n* = 53).

| Characteristics | H-score = 0  (*n* = 20) | H-score > 0  (*n* = 33) | *p-*value |
| --- | --- | --- | --- |
| Age  < 65 years  ≥ 65 years | 13  7 | 19  14 | 0.59 |
| Gender  Female  Male | 8  12 | 12  21 | 0.79 |
| WHO grade  Grade 3  Glioblastoma | 5  15 | 2  31 | 0.09 |
| Origin  De novo  Secondary | 18  2 | 30  3 | 1.00 |
| Primary vs. recurrent  Primary  Recurrent | 15  5 | 28  5 | 0.48 |
| Multiplicity  Single  Multiple | 18  2 | 24  9 | 0.18 |

Abbreviations: IL-13Rα2, interleukin-13 receptor alpha 2
